# The Efficacy of Adalimumab as an Initial Treatment in Patients with Behçet’s Retinal Vasculitis

**DOI:** 10.3389/fphar.2021.609148

**Published:** 2021-06-22

**Authors:** Shizhao Yang, Zhaohao Huang, Yunwei Hu, Jian Zhang, Xiuxing Liu, He Li, Lihui Xie, Feng Wen, Dan Liang, Wenru Su

**Affiliations:** State Key Laboratory of Ophthalmology, Zhongshan Ophthalmic Center, Sun Yat-sen University, Guangzhou, China

**Keywords:** adalimumab, behçet's uveitis, retinal vasculitis/diagnosis, initial treatment, naïve

## Abstract

**Background:** No study has evaluated the effectiveness of Adalimumab (ADA) as first-line in treatment-naïve patients with retinal vasculitis due to Behçet’s Uveitis (BU).

**Objective:** To compare the efficacy of ADA plus conventional therapy and conventional therapy alone as initial treatments in naïve BU patients characterized by retinal vasculitis.

**Methods:** Medical records of BU patients characterized by retinal vasculitis treated with conventional therapy (CT, refers to glucocorticoid and immunosuppressive agents) alone or ADA plus conventional therapy with at least 6 months of follow-up between February 2015 and June 2020 were analyzed. Only patients who were first diagnosed with BU without previous systemic treatment were reviewed. The retinal vasculitis score based on fluorescein angiography (FA), best-corrected visual acuity, glucocorticoid-sparing effect, the number of relapses and ocular complications were evaluated.

**Results:** A total of 45 patients (87 eyes) were included. Twenty-four patients (55.33%) in the CT group were treated with conventional therapy and 21 patients (46.67%) in the ADA group were treated with ADA plus conventional therapy. The inflammatory parameters improved in both groups. FA scores showed significantly greater improvement in ADA group than CT group (*p* < 0.001). The median number of relapses was significantly lower, and the duration of remission was longer in ADA group than CT group (*p* < 0.001). At the last visit, a significantly better BCVA improvement (*p* = 0.024), better inflammation control (anterior chamber inflammation *p* = 0.017 and vitritis *p* < 0.001) and lower daily glucocorticoid dosage (*p* = 0.005) were identified in patients received ADA therapy. In CT group, 1 patient suffered hepatitis B and tuberculosis, 1 had growth retardation, 1 patient had with osteoporosis, then followed by other mild AEs (mostly respiratory upper tract infections); while in ADA group, 1 patient experienced a mild pneumonia (*n* = 1) while milder AEs were represented mostly by respiratory upper tract infections followed by gastrointestinal discomfort.

**Conclusion:** ADA plus conventional therapy achieved superiority over conventional therapy as initial treatment in naïve BU patients with retinal vasculitis.

## Introduction

Behçet’s disease (BD) is a multisystem vasculitis disorder of uncertain aetiology with a series of manifestations, including recurrent oral ulcers, genital ulcers, dermic lesions and uveitis. Although the incidence of BD is not very high (15–420 cases per 100,000 population in the area along the ancient Silk Road and 0.12–0.33 cases per 100,000 population in North America), ocular involvement appears to be one of the most common devastating manifestations of BD (approximately 50–70%) (Fabiani et al., 2017; Tai et al., 2017; Khabbazi et al., 2019; Sota et al., 2020; Yalçindag and Köse, 2020). Several studies indicated that the risk of severe visual loss significantly increased when the ocular posterior segment was involved (Pivetti Pezzi et al., 1985; Benezra and Cohen, 1986; Kitaichi et al., 2007; Yang et al., 2008). Therefore, the rapid suppression of inflammation and prevention of recurrence are essential to avoid irreversible ocular damage and permanent blindness (Ozyazgan et al., 2015). As a conventional treatment, Behçet’s uveitis (BU) is treated systemically with high doses of corticosteroids in conjunction with at least one immunosuppressive agent, including cyclosporine (CsA), azathioprine (AZA), methotrexate (MTX) and mycophenolate mofetil (MMF), while topical corticosteroids and mydriatic drops are used when inflammation of the ocular anterior segment is present (Jabs et al., 2000). Unfortunately, approximately 25% of those patients become blind even with consecutive conventional treatment. Furthermore, a series of side effects induced by high-dose and long-term corticosteroids are inevitable (Oray et al., 2016; Fabiani et al., 2017).

Substantial evidence has suggested that tumor necrosis factor-α (TNF-α) is the primary cytokine involved in the pathogenesis of BU (Ahn et al., 2006; Abu El-Asrar et al., 2019). Counteracting TNF-α with a TNF-α inhibitor is a promising strategy for the treatment of BU. Adalimumab originator, a fully humanized monoclonal antibody specifically targeting TNF-α, has been proven to have ideal efficacy in a series of rheumatic diseases, including BD. Furthermore, the indication of non-infectious uveitis was approved by the US Federal Drug Administration (FDA) and European Medicines Agency (EMA) in 2016. Several studies have demonstrated the efficacy of ADA in BU in France, Spain, and Italy (Ahn et al., 2006; Perra et al., 2012; Vallet et al., 2015, 2016; Fabiani et al., 2018). However, researchers have mainly focused on refractory BU patients who have inadequate responses to conventional treatment and suffer from irreversible vision damage. Few researchers have focused on the efficacy of ADA as an initial approach in the treatment of naïve BU patients who had not been treated systemically before.

Notably, retinal vasculitis, the major risk of vision impairment, (Abu El-Asrar et al., 2005; Ali et al., 2014; Sharief et al., 2017), is the most severe ocular lesion and can be found in up to 33.6% of BU patients (Davatchi et al., 2016). Macular edema (ME) in the early stage and optic nerve atrophy and diffuse retinal atrophy in the terminal stage, resulting from long-lasting and uncontrolled retinal vasculitis, are the fundamental vision-threatening pathologies of BU (Ali et al., 2014). Thus, eliminating retinal vasculitis is one of the significant objectives in the management of BU. However, current studies of ADA have not adequately focused on retinal vasculitis.

The main objective of this retrospective study was to compare the efficacy and safety of ADA plus conventional therapy with those of conventional therapy alone as an initial treatment for BU patients who had not been treated systemically before. Retinal vasculitis as the fundamental impairment was the primary indicator in a quantified method in our study.

## Methods

### Patient Selection

A retrospective, comparative study was conducted in BU patients treated at Zhongshan Ophthalmic Center between February 2015 and April 2021. This study was performed according to the principles of the Helsinki Declaration and was approved by the ethics committee of Zhongshan Ophthalmic Center. Written informed consent was acquired from each patient.

To be included, the patients had to 1) meet the International Study Group for Behçet’s Disease (ISG) or International Criteria for Behçet’s Disease (ICBD); 2) present with uveitis with retinal vasculitis; 3) receive no systemic anti-inflammatory treatment; 4) have regular follow-ups at least 6 months after initial treatment; and 5) have two or more examinations with fluorescein angiography (FA) at the first and last visits. Patients were divided into two therapeutic groups depending on whether ADA was used or not. The conventional therapy group (CT group) included patients who were treated with oral glucocorticoids plus immunosuppressive agents, and the ADA group included those who resorted to the combination of ADA and immunosuppressive agents with oral glucocorticoids.

Patients were excluded 1) if they had an episode of persistent duration for at least 3 months from onset; 2) if they had a history of systemic disease controlled by long-term medication except for BD; or 3) if the refractive medium was too opaque to identify a clear image on FA and optic coherence tomography (OCT) or other ocular examinations.

### Data Collection

Individual data were collected and analyzed retrospectively, including demographic characteristics, therapeutic management and ocular parameters. The primary outcome was the cumulative change from baseline in FA score of retinal vasculitis. The secondary outcomes consisted of cumulative changes in visual acuity, anterior chamber cell (ACC) grade, vitritis grade, macular thickness (MT) and ocular complications. The cumulative changes were defined as post-treatment values subtracted from pre-treatment values. In addition, the number of relapses, glucocorticoid-sparing effects, systemic disorders and adverse events (AEs) were recorded for each patient.

*Best-Corrected Visual acuity (BCVA)* BCVA was evaluated according to the Snellen chart. Extremely low visual acuity classified by a semiquantitative scale as “counting finger/hand motion/light perception” was converted to a quantified visual acuity value for statistical convenience (Schulze-Bonsel et al., 2006).

*Anterior chamber cell (ACC) grade and vitritis grade* The ACC grade was evaluated with the use of a slit-lamp biomicroscope and recorded according to recommendations of the Standardization of Uveitis Nomenclature (SUN) Working Group in 1990 (Supplementary Table 1) (Jabs et al., 2005). The degree of vitreous haze was evaluated by the Nussenblatt scale (Supplementary Table 2) (Nussenblatt et al., 1985).

*Macular thickness (MT)* MT measurements were performed by a Cirrus HD-OCT (Carl Zeiss, CA, United States) and obtained by the 512 × 128 scan pattern. Macular thickening was defined as MT >250 μm, while ME was classified as MT >300 μm.

*FA scores of retinal vasculitis* Signs of retinal vasculitis referred to vascular leakage, staining and occlusion, which can be assessed by FA. Assessment of FA scores of retinal vasculitis was based on the scoring system issued by the Angiography Scoring for Uveitis Working Group (ASUWOG) (Supplementary Table 3) (Tugal-Tutkun et al., 2010). To minimize the deviation, FA scores were evaluated separately by 2 specialists (YSZ and HZH) and double-checked to ensure no obvious error. In cases of a disagreement, a decisive verdict was given by senior specialist SWR.

*Ocular complications* These included but were not limited to 1) eye pain, eye discharge or eye swelling, 2) dry eye or other disorders of the ocular surface, 3) intraocular pressure increase, 4) a new-onset cataract or worsening cataract requiring surgery, and 5) any other symptoms not associated with BD.

*The number of relapses* Remission was defined as any sign of intraocular inflammation that vanished for at least 3 months. A relapse was defined as a new flare of uveitis (such as anterior or posterior chamber inflammation, retinal vasculitis, papillitis or macular edema) in a patient who was in remission.

*Glucocorticoid-sparing effect* ADA and immunosuppressive agents are used to reduce the duration of glucocorticoid therapy. The evaluation parameter of the glucocorticoid-sparing effect was a reduction in glucocorticoid usage.

*System disorder monitoring* Routine blood examination, liver and renal function, and electrocardiogram were monitored during treatment. Any system disorder was recorded and analyzed.

*Adverse events* AEs included but were not limited to 1) new-onset or reactivated infections, 2) gastrointestinal discomfort, 3) injection site or allergic reactions, and 4) immunogenicity-related AEs. Adverse events were recorded from the receipt of the first dose of ADA and reported regardless of severity or perceived association with ADA.

### Statistical Analysis

In the descriptive analysis of baseline characteristics, categorical variables were reported as frequency and percentage. The Shapiro–Wilk test was applied to assess the normality assumption for continuous variables. Normally distributed variables and non-normal variables were reported as mean (SD) and median (IQRs), respectively. Demographic and clinical characteristics between two groups were compared using Chi-square test, Student’s *t*-test or Mann–Whitney *U* test, as appropriate. The primary outcome and secondary outcome between groups were compared using mixed-effect models to account for the correlation between two eyes within the same subject in this manuscript, with characteristics not fully balanced between group as covariate, prespecified as *p* < 0.20 for baseline comparisons. Serologic abnormities, extra-ocular manifestations and adverse events were recorded and listed. All analyses were performed using SAS version 9.4 (SAS Inc., Cary, NC, United States) with significance at *p* < 0.05.

## Results

### Baseline Demographics of the Different Groups

We retrieved 158 medical records of BU patients with retinal vasculitis who were admitted to our clinic at Zhongshan Ophthalmic Center between February 2015 and June 2021. After excluding patients without complete records (*n* = 30) or those who received systemic treatment before referral to us (*n* = 56), 72 records were identified and examined in detail. We chose patients who had at least a 6-months treatment period and more than 2 FA checks (Flow chart presented in Supplementary Figure 1)

Finally, a total of 45 patients (87 eyes) were included in this study. Among the patients included, 21 out of 45 patients (42 eyes) resorted to ADA therapy (46.67%), whereas 24 (45 eyes) proceeded with a conventional therapeutic regimen (53.33%). Both groups had equal sex distributions (*p* = 0.967). The mean age in the CT group was slightly older than that in the ADA group, with no significant difference (*p* = 0.107). Most patients in both groups had bilateral uveitis (87.5% in the CT group and 100% in the ADA group). The mean duration of the disease course prior to the initiation of the study intervention and the mean follow-up duration were similar in both groups, with no significant difference.

All demographic data are presented in Table 1.

**TABLE 1:** Demographic characteristics of 45 treatment-naïve patients with BU with retinal vasculitis who received ADA or conventional therapeutic regimen.

	CT (*n* = 24)	ADA (*n* = 21)	*p*
Patients/eyes	24/45	21/42	–
Age, mean ± SD years	26.54 ± 10.47	22.24 ± 15.30	0.107
Sex, males/females	9/15	8/13	0.967
Duration of previous disease course, median (IQR) weeks	2 (1, 8)	2 (0, 5.5)	0.550
Treatment period, median (IQR) months	17.00 (12.25, 22.00)	18 (13, 18.5)	0.722

CT, conventional treatment; ADA, adalimumab; IQR, interquartile range; BCVA, best-corrected visual acuity. *p < 0.05.

### Ocular Parameters at Baseline in Different Groups

The ocular parameters were comparable at baseline in the two groups and are presented in Table 3. ACC grade was approximately at same level in both groups [1.27(95% CI, 0.87–1.67) in CT group, 1.51(95% CI, 1.09–1.93) in ADA group, *p* = 0.411], while vitritis grade in the CT group was lower than that in the ADA group [0.94(95% CI, 0.68–1.19) in the CT group, 1.37(95% CI, 1.09–1.64) in the ADA group, *p* = 0.027]. BCVA [0.47 (95% CI, 0.37–0.57) in the CT group, 0.42 (95% CI, 0.31–0.52) in the ADA group] and MT [395.00 (95% CI, 319.19–470.80) μm in the CT group, 347.95 (95% CI, 267.59–428.30) μm in the ADA group] were similar at baseline (*p* > 0.1). FA scores were similar at baseline between the groups [21.47 (95% CI, 19.57–23.36) points in the CT group, 19.90 (95% CI, 17.91–21.90) points in the ADA group] (*p* = 0.259).

### Therapeutic Management in Different Groups

Glucocorticoids were given in the two groups at a dose of 0.8–1.0 mg/kg/d at the first visit and tapered according to the symptoms and severity of disease at the follow-ups. In the CT group, the immunosuppressive agents were MTX or CsA alone or combined with CTX, AZA or MMF. Thalidomide was used for 1 patient in the CT group. In the ADA group, all patients received ADA therapy with a loading dose of 80 mg initially and a subsequent dose of 40 mg subcutaneously every other week. Co-medication with immunosuppressive agents was initiated with MTX or CsA alone or in combination with CTX, AZA or MMF. Other treatments were given based on certain conditions: 1) periocular triamcinolone acetonide and subconjunctival injection of dexamethasone were given to 9 patients from the CT group and 14 patients from the ADA group with severe anterior segment inflammation, severe retinal vasculitis and macular edema; 2) intravitreal injection of conbercept (Lumitin, Chengdu) was given to 1 patient from the CT group and 2 patients from the ADA group with refractory macular edema or neovascularization (lasting over 3 months); 3) all 45 patients were initiated with prednisolone acetate (Pred Forte, Allergan, Dublin) or tobramycin dexamethasone (Tobradex, Alcon, Fort worth, Texas) due to anterior chamber inflammation and decreased the frequency, switched to weaker glucocorticoid droplets or withdrew as inflammation was alleviated; and 4) all 45 patients received atropine ointment or tropicamide droplets due to chamber inflammation.

There was no significant difference in oral prednisone use between groups (100% in the ADA group and 100% in the CT group), and the mean daily dosage of initial glucocorticoid treatment was 53.11 ± 11.45 mg/d vs. 55.24 ± 11.94 mg/d in the CT group and ADA group, respectively (*p* = 0.399). Intravenous methylprednisolone was used in patients with acute and severe intraocular inflammation characterized by ACC or vitreous cell grade 3+ to 4+ (25.00% in the CT group and 42.86% in the ADA group, *p* = 0.226; mean daily dosage: 112.73 ± 16.18 mg/d vs. 124.44 ± 12.94 mg/d in the CT group and ADA group, respectively, *p* = 0.057). The choice of conventional immunosuppressive therapy for patients was similar in both groups (presented in Table 2). No significant difference was observed between groups in the prescription of immunosuppressive agents.

**TABLE 2:** Category and dosage of treatments of patients in different groups at baseline.

	CT (*n* = 24)	ADA (*n* = 21)	*p*
Systemic treatments at baseline, patients (*n*, %)			
Oral glucocorticoid	24 (100%)	21(100%)	–
IV Methylprednisolone	6 (25.00%)	9(42.86%)	0.226
CsA	13 (54.17%)	21 (100%)	<0.001*
MTX	19 (79.17%)	21 (100%)	0.051
MMF	1 (4.17%)	0 (0%)	1.000
CTX	7 (29.17%)	11 (52.38%)	0.138
Others^a^	1	0	–
Dosage at baseline, mean ± SD			
Oral glucocorticoid, mg qd	53.11 ± 11.45	55.24 ± 11.94	0.399
IV Methylprednisolone, mg qd	112.73 ± 16.18	124.44 ± 12.94	0.057
CsA, mg bid	80.77 ± 26.75	84.52 ± 29.71	0.592
MTX, mg qw	15.35 ± 11.97	16.31 ± 4.03	0.625
MMF, g bid	–	–	–
CTX, g qd	0.42 ± 0.09	0.45 ± 0.16	0.572
Local treatments at baseline, patients (*n*, %)			
Glucocorticoid droplet	24 (100%)	21 (100%)	–
Others^b^	15 (62.50%)	17 (80.95%)	0.205
Prednisone at final visit, patients (*n*, %)	20 (83.3%)	11 (53.4%)	0.051
Prednisone dosage at the final visit, mean ± SD, mg/d	22.63 ± 20.83	7.50 ± 2.96	0.005*
Prednisone dosage reduction, mg	33.65 ± 20.50	51.31 ± 11.45	0.001*
Relapse, median (IQR)	3 (1, 4.5)	0 (0, 1)	<0.001*

Abbreviations: IV, intravenous injection; CsA, cyclosporin A; MTX, methotrexate; CTX, cyclophosphamide; MMF, mycophenolate mofetil.

*p < 0.05. ^a^Other systemic treatments included thalidomide in 1 patient in the conventional treatment group. ^b^Others: CT group: periocular injection of triamcinolone acetonide in 11 patients, subconjunctival injection of dexamethasone in 2 patients, intravitreal injection of conbercept in 1 patient and anti-hyper-intraocular treatment in 1 patient. ADA group: periocular injection of triamcinolone acetonide in 14 patients, conbercept in 2 patients and anti-hyper-intraocular treatment in 1 patient.

### Ocular Parameter Improvement in Different Groups at the Last Visit

Ocular parameters at the last visit were compared with those at baseline, and the differences in changes between the two groups were analyzed and are presented in Table 3.

**TABLE 3:** Clinical features of 87 eyes with retinal vasculitis before and change in clinical features after receiving different regimens.

	Baseline, mean (95CI%)				Change, mean (95% CI)			
	CT (*n* = 45)	ADA (*n* = 42)	*p*	Difference (95% CI)	CT (*n* = 45)	ADA (*n* = 42)	*p*	Difference (95% CI)
BCVA	0.47(0.37–0.57)	0.42(0.31–0.52)	0.484	0.05(−0.10–0.20)	0.06(−0.06–0.18)	0.33(0.21–0.46)	0.024*	0.28(0.11–0.45)
ACC grade	1.27(0.87–1.67)	1.51(1.09–1.93)	0.411	−0.24(−0.83 to 0.34)	−0.38(−0.85 to 0.09)	−1.22(−1.70 to −0.74)	0.017*	−0.84(−1.52 to −0.16)
Vitritis grade	0.94(0.68–1.19)	1.37(1.09–1.64)	0.027*	−0.43(−0.81 to −0.05)	−0.06(−0.45 to 0.33)	−1.37(−1.78 to −0.97)	<0.001*	−1.32(−1.88 to −0.75)
MT, μm	395.00(319.19–470.80)	347.95(267.59–428.30)	0.387	47.05(−61.68–155.79)	−102.90(−191.80 to −14.01)	−137.78(−224.52 to −51.03)	0.576	−34.88(−160.24 to 90.49)
FA score	21.47(19.57–23.36)	19.90(17.91–21.90)	0.259	1.56(−1.19–4.32)	−3.75(−6.35 to −1.15)	−12.85(−15.61 to −10.08)	<0.001*	−9.10(−12.92 to −5.27)

Abbreviation: BCVA, best −corrected visual acuity, ACC, Anterior chamber cell, MT, macular thickness, FA, fluorescein angiography, CI, confidence interval.

*p < 0.05. The primary outcome was the cumulative change from baseline in FA score of retinal vasculitis. The secondary outcomes consisted of cumulative changes in BCVA, ACC grade, vitritis grade, MT. Changes in clinical features are defined as post−treatment values subtracted from pre-treatment values. All means were derived from mixed models.

**FIGURE 1:**
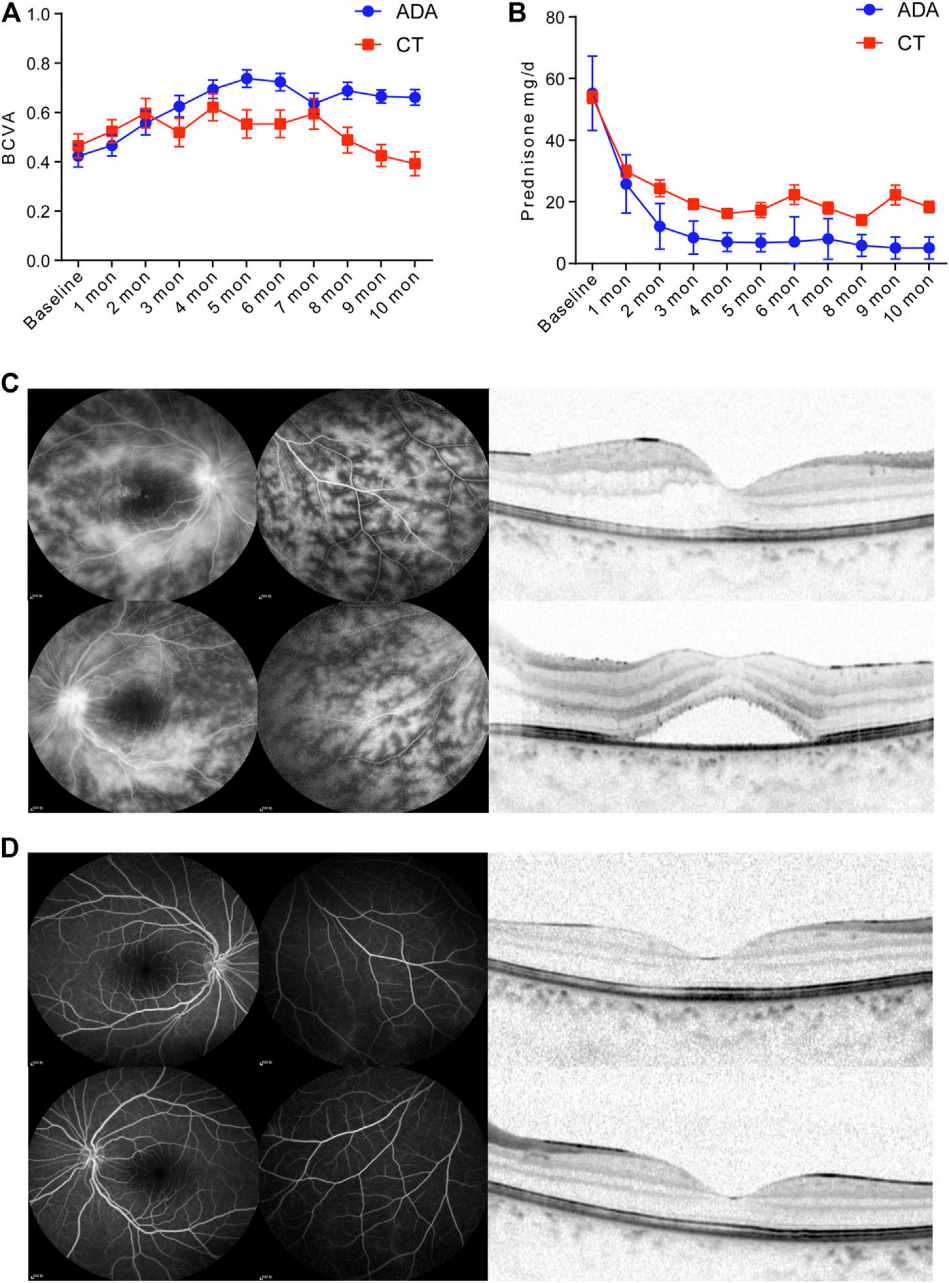
**(A)** BCVA of treatment-Naïve patients with BU retinal vasculitis in the CT group and ADA group at monthly regular visits. **(B)** Daily glucocorticoid dosage of patients in the CT group and ADA group at monthly regular visits and at final follow-up. In CT group, 20 (83.3%) patients proceeded oral glucocorticoid with dose of 22.63 ± 20.83 mg/d, while in ADA group, 11 (53.4%) patients proceeded oral glucocorticoid with dose of 7.50 ± 2.96 mg/d. (C) A 10-year-old female BU patient with a BCVA of 0.4 (OD) and 0.16 (OS). FA imaging (10 min) showed **(C-left panel)** severe “fern-like” pattern change and **(C-right panel)** OCT scan observed edema in the macula with thicknesses of 277 and 483 μm in the right (top) and left (bottom) eyes, respectively. **(D-left panel)** FA imaging (10 min) of the same patient after ADA administration at the 5-months follow-up shows signs of vasculitis disappearance. The BCVA of the patient improved to 0.63 (OD) and 0.63 (OS). **(D-right panel)** Normalization of morphology in macular region in OCT scan after ADA treatment.

The patients in the ADA group [the BCVA improved by 0.33 (95% CI, 0.21–0.46)] gained an increased visual benefit compared with those in the CT group [the BCVA improved by 0.06 (95% CI, −0.06 to 0.18)] (*p* = 0.024). The BCVA was obviously improved in both groups during the first 2 months after treatment. It experienced a rising trend and remained at a satisfactory level of visual acuity in the ADA group, even though it decreased slightly at 7 months after treatment. However, the BCVA of the CT group fluctuated during treatment. There was no improvement at the last visit. After 5 months of treatment, a significantly better BCVA was achieved in the ADA group than in the CT group (Figure 1A).

Decreases in ACC grade and vitritis grade were detected in both groups, but the ADA group exhibited a better improvement in anterior and posterior chamber inflammation than the CT group (*p* = 0.017 in anterior inflammation and *p* < 0.001 in vitritis). The decrease in MT was lower in the CT group (−102.90[95% CI, −191.80 to −14.01] μm) than in the ADA group (−137.78[95% CI, −224.52 to −51.03] μm), but the difference was not statistically significant (*p* = 0.576). Detailed information is provided in Table 3.

The improvement of FA scores in the ADA group was distinctly larger than that in the CT group at the last visit [−3.75(95% CI, −6.35 to −1.15) points in CT group; −12.85 (95% CI, −15.61 to −10.08) points in ADA group] (*p* < 0.001). The improvement of FA scores primarily took place in capillary leakage.

Ocular complications in both groups were recorded as follow: 1) In the CT group, 16 patients (28 eyes) had newly onsetting or advancing of complicated cataract in various degree, and 2 of them (4 eyes) received cataract surgery; 1 patient (1 eye) had secondary central serous chorioretinopathy and restored after discontinuing glucocorticoids; a temporary elevation of intraocular pressure over 21 mmHg was found in 2 patients (2 eyes) and 2 patient (4 eyes) had epiretinal membrane. 2) In the ADA group, 12 patients (21 eyes) had newly onsetting or advancing of complicated cataract in various degree, and 1 of them (2 eyes) received cataract surgery; 1 patient (2 eye) had secondary central serous chorioretinopathy and restored after replacing glucocorticoids with tofacitinib; 2 children had band-shape keratopathy; a temporary elevation of intraocular pressure over 21 mmHg was found in 6 patients (6 eyes).

### Relapse, Glucocorticoid-Sparing Effect, System Disorder Monitoring and Adverse Events in Different Groups

Patients in the ADA group received an average of 16.05 ± 6.79 injections of ADA, including 1 patient who received 2 injections of an ADA biosimilar, Qletli (Bio-Thera, Jiangsu, China), due to the COVID-19 quarantine in China.

In the CT group, the median number of relapses was 3 (1, 4.5) times during the treatment period, while in the ADA group, the mean number of relapses was 0 (0, 1). The difference was statistically significant (*p* < 0.001).

After the initial treatment, the dosage of daily glucocorticoids presented a more distinct decrease among patients in the ADA group than among those in the CT group from 1 month to 10 months (Figure 1B), with a significant difference. As expected, in the remaining patients who still received oral glucocorticoids treatment (20 patients in CT group and 11 patients in ADA group) in the final visit, there was a significant difference in the mean daily dosage of glucocorticoids between the two groups at the last visit (mean dosage of 22.63 ± 20.83 mg/d in the CT group vs. 7.5 ± 5.96 mg/d in the ADA group) (*p* = 0.005).

The reports of serological tests (including routine blood examination and hepatorenal function) and electrocardiograms were checked to evaluate systemic disorders. Some patients experienced a slight abnormality, as listed in Table 4.

**TABLE 4:** Serologic abnormalities in patients from different groups.

Main abnormality	CT (*n* = 24)	ADA (*n* = 21)
Routine blood	8	12
Hepatorenal function	7	8
Coagulation function	3	1
ESR	3	2
ECG	0	0

Abbreviation: ESR, erythrocyte sedimentation rate, ECG, electrocardiogram.

Routine blood abnormalities included counting and percentage of leukocytes/erythrocytes/platelets and other parameters.

Hepatorenal function abnormalities included abnormal values or ratios of alanine aminotransferase/aspartate aminotransferase, abnormal values or ratios of albumin/globulin, total cholesterol, triglyceride, creatinine and other parameters.

Coagulation function abnormalities included abnormal activated partial thromboplastin time (APTT), prothrombin time (PT) and other parameters.

Adverse events in both groups were recorded as follows: 1) In the CT group, a patient had hepatitis B and tuberculosis and then suspended oral glucocorticoids; growth retardation was found in 1 patient and osteoporosis was detected in 1 patient. Other minor AEs included respiratory disorders, gastrointestinal and others. 2) In the ADA group, infection was found in 6 patients, including 5 with upper respiratory infection and 1 with mild pneumonia (which presented as dryness of throat, fever with temperature of 38.3°C and patchy shadows on computed topography scans). After delaying the injection of ADA, upper respiratory infection symptoms in 5 patients quickly disappeared. After roughly 1 week of oral antibiotic treatment, the temperature and computed topography scans image of the patient who suffered pneumonia in ADA group normalized. Some patients reported respiratory disorders and gastrointestinal disorders. No patient discontinued ADA treatment due to severe adverse events. Detail information about extra-ocular manifestations and AEs were introduced in Table 5.

**TABLE 5:** Extra-ocular manifestations of BD and drug-related AEs before and after the treatment.

	Before treatment		After treatment	
Main abnormality	CT (*n* = 24)	ADA (*n* = 21)	CT (*n* = 24)	ADA (*n* = 21)
Oral ulcer	15	15	3	1
Genital ulcer	0	0	0	0
Central nervous system symptoms	0	0	0	0
Vascular involvements	0	0	0	0
Skin erythema, rash or other skin changes	3	3	4	2
Articular symptoms	3	0	0	1
Upper respiratory infection or fever	0	0	3	7
Gastrointestinal symptoms	0	0	0	2
Hepatitis B	0	0	1	0
Tuberculosis	0	0	1	0
Pneumonia	0	0	0	1
Insomnia	0	0	0	1
Others	0	0	2	0

Other skin changes including: In CT group, pox in 1 patient, alopecia in 1 patient and hydroxychloroquine-related skin change in 1 patient who suffered Hepatitis B; in ADA group, dermatophyte in 1 patient and alopecia in 1 patient. Glucocorticoid-related acne was not included in skin changes.

Other situations including: In CT group, 1 patient complained about growth retardation, and 1 patient complained about osteoporosis.

## Discussion

In our study, good clinical effectiveness, tolerability and safety of ADA were achieved in the initial treatment of naïve BU patients with retinal vasculitis. The patients in the ADA group gained more visual benefits and increased improvement of intraocular inflammation than those in the CT group. The dosage of glucocorticoids decreased due to ADA therapy. Moreover, systemic disorders and adverse events were not common during treatment.

BU is one of the most common forms of uveitis in the Far Eastern area, with a prevalence ranging from 5.8 to 32.2% (Chung and Choi, 1989; Kotake et al., 1997; Chang and Wakefield, 2002; Wakabayashi et al., 2003). In China, Yang et al*.* reported that BU was the predominant type of uveitis with a proportion of 16.5%, and it is also the leading cause of blindness among Chinese uveitis patients (Yang et al., 2005). Although the precise pathogenesis of BU remains unknown, diffuse obliterative retinal vasculitis is recognized as the essential pathology. Chronic damage in retinal structures secondary to retinal vasculitis can significantly impair visual prognosis due to macular edema in the early stages and optic nerve atrophy, diffuse retinal atrophy and occlusion of retinal vessels with subsequent retinal fibrosis in a more advanced disease (Yang et al., 2008). Frequently, impairments are irreversible and profoundly correlated with prognosis, especially when the region within the retinal vascular arcade is involved. Antiretinal antibodies derived from the damaged retinal barrier, oxidative stress and apoptotic death of retinal photoreceptors caused by inflammatory mediators and macular ischaemia have been suggested as potential explanations for poor visual outcome in BU patients (Rao and Wu, 2000; Liversidge et al., 2002; Yilmaz et al., 2002; Rajendram et al., 2007).

As mentioned above, sufficient initial treatment at an early stage of the disease course is extremely significant. Unfortunately, there is no consensus about the standard therapeutic regimen for treatment-naïve BD patients with retinal vasculitis. Currently, the mainstay of treatment is a combination therapy of high-dose systemic corticosteroids and immunosuppressive agents, including CsA, AZA and MTX. However, glucocorticoids are initiated frequently at a rather high dosage, and long-term sustained treatment is demanded (normally no less than one year), which may create a dilemma between inadequate response and a series of side effects (Wei et al., 2004; Huscher et al., 2009; Thomas, 2019).

Basic studies have provided a strong biologic rationale for the TNF-α blockade in BU which may result in both a rapid disease remission and a sustained response (Ahn et al., 2006; El-Asrar et al., 2011; Fabiani et al., 2019). ADA, a fully humanized monoclonal antibody specifically targeting TNF-α, has been proven to be a feasible and prospective option for the treatment of non-infective uveitis and has been approved by the FDA and National Medical Products Administration (NMPA) in the treatment of non-infectious uveitis in 2016 and 2020, respectively.

In 2012, a randomized controlled multicentre trial in Spain and Latin America (including Mexico and Venezuela) reported a favourable effectiveness of ADA in 131 patients with non-infectious uveitis, but only 13 of the patients were diagnosed with BU. No details about the number of affected eyes or uveitis patterns were provided, nor were any specific follow-up data provided (Díaz-Llopis et al., 2012). In 2014, a multicentre study with 124 patients who were diagnosed with BU revealed that clinical control of inflammation was achieved in 84 of 124 patients using anti-TNF agents (67.7%) (Calvo-Río et al., 2014). In addition, retinal vasculitis was initially found in only some of the patients, and the outcomes of retinal vasculitis after the treatment were roughly summarized as the presence or absence of retinal angiographic leakage. Fabiani et al. (2017) reported that ADA provides long-term control of ocular inflammation in patients with BU. The same researchers also investigated the efficacy of ADA in patients with retinal vasculitis (Fabiani et al., 2018). Though the efficacy of ADA in retinal vasculitis seemed inspiring, the primary disease of their patients was not solely confined to BU, and patients with spondylarthritis, Crohn’s disease and some unmentioned diseases were also included. In Asia, sporadic studies have reported similar outcomes, including Far Eastern areas such as Japan and Hong Kong (Bawazeer et al., 2010; Al-Fakhri and Alsulaiman, 2019; Hiyama et al., 2019; Ho et al., 2019).

Hitherto, most reported studies were case series of ADA treatment in refractory BD-associated uveitis or retrospective controlled studies among different anti-TNF agents in the treatment of non-infectious uveitis. Here, we presented the first study to compare the effectiveness and safety between ADA plus conventional therapy and conventional therapy alone in naïve patients with BU. After more than 6 months of treatment, improvements in ocular inflammation (such as ACC grade and vitritis grade), visual outcomes, macular morphology, retinal vasculitis and relapse frequency were more significant in the ADA group than in the CT group, which is consistent with previous studies, indicating that better efficacy was achieved in the ADA group. For glucocorticoid-sparing effects, the dose of glucocorticoids tapered by a large margin compared with conventional therapy. The adverse events occurred under control conditions. Hence, ADA might be a feasible alternative to minimize the risks of long-term glucocorticoid usage.

Notably, a prominent superiority was unveiled in the ADA group compared with the conventional therapeutic group in controlling retinal vasculitis by introducing a quantified scoring system to assess the severity of vasculitis. In previous studies, retinal vasculitis was evaluated by a binary grading system, with or without the presence of evidence of active vasculitis. This ambiguous classification of retinal vasculitis can be biased in the evaluation of ADA efficacy. This scoring system could reduce the bias because ameliorating FA leakage can be an indicator of effectiveness as well. In our study, retinal vascular and capillary leakage, especially within the retinal vascular arcade and macula region, was ameliorated dramatically in most patients in the ADA group, whereas only slight changes occurred in most patients in the CT group. Most importantly, due to the natural course of BU, rapid suppression of inflammation and prevention of recurrence were essential in the early stage, which led to a more promising visual outcome. Although the recommendation of the American Academy of Ophthalmology in 2014 and the European League Against Rheumatism (EULAR) in 2018 indicated that ADA could be used as first-line therapy in eye involvement of BD, research on ADA as an initial treatment in the management of BU remains scarce. In most scenarios, ADA was mainly used as a second-line therapy when the combination of glucocorticoid plus immunosuppressive agent therapy produced a dissatisfactory outcome. Our study filled this knowledge gap in ADA treatment in treatment-naïve patients and indicated that ADA outcompeted conventional therapy in these patients.

A minor possibility of adverse events, including malignancy, inflammatory neurologic disease, opportunistic infections, reactivation of latent tuberculosis, and congestive heart failure, has been reported in patients who receive ADA treatment (Keane et al., 2001; Diak et al., 2010; Solomon et al., 2011; Ruderman, 2012). In this study, only 1 severe event in the ADA group was detected, with 1 patient suffering from pneumonia. However, some minor adverse events were found in both groups. The most common adverse events in the ADA group were minor infection, respiratory disorder and gastrointestinal disorder. One patient complained about growth retardation, and 1 patient complained about osteoporosis in the CT group. No patient discontinued treatment due to adverse events in the ADA group. The frequency of minor adverse events was slightly higher in the ADA group. Hence, continuous monitoring is required for the sake of safety. Once adverse events happen, ADA treatment should be suspended until the adverse events are completely controlled. No severe adverse events were found during the treatment in the ADA group except for 1 patient suffered from mild pneumonia, but it requires a long observation time and a large sample size to assess the long-term risk of severe adverse events.

## Limitations

To our knowledge, this study is the first to compare the efficacy of ADA therapy in treatment-naïve patients with BU. Moreover, this is the first study to explore the efficacy of ADA in treatment-naïve patients with retinal vasculitis due to BU by using a quantified scoring system. However, limitations are unavoidable. First, since this study was a retrospective study, inherited shortcomings and biases of retrospective study was unavoidable. Second, owning to its rarity, relatively small sample size and homogeneity of population (mainly Chinese Han) of this cohort, which hindered the generalization of our findings in other ethnic groups. Further study with larger cohort and longer observation is urgently needed to better assess retinal vasculitis in BU.

## Conclusion

Our results indicate that superior efficacy was achieved in treatment-naïve patients with BU characterized by retinal vasculitis who received ADA as initial treatment compared with conventional therapy.

## Data Availability

The original contributions presented in the study are included in the article/Supplementary Material, further inquiries can be directed to the corresponding authors.
