# Supplementary material for: The Efficacy of Adalimumab as an Initial Treatment in Patients with Behçet’s Retinal Vasculitis
Source: Front Pharmacol. 2021 Jun 22;12:609148. doi: 10.3389/fphar.2021.609148 (PMC8258106; doi:10.3389/fphar.2021.609148)
Supplement: Supplementary file 1 [file DataSheet1.docx]

**Supplementary Table 1. The SUN Working Group Grading Scheme for Anterior Chamber Cells**

| Grade | Cells in Field |
| --- | --- |
| 0 | <1 |
| 0.5+ | 1-5 |
| 1+ | 6-15 |
| 2+ | 16-25 |
| 3+ | 26-50 |
| 4+ | >50 |

**SUN=Standardization of uveitis nomenclature. Field size is 1 mm×1 mm slit beam.**

**Supplementary Table 2. The Nussenblatt Scale for Vitreous Haze**

| Grade | Clarity of fundus landmarks |
| --- | --- |
| 0 | No evident vitreal haze at all |
| 1+ | Better definition of both optic nerve head and retinal vessels than 2+ |
| 2+ | Better visualization of retinal vessels than 3+ |
| 3+ | Optic nerve head can be observed, but the borders are quite blurry |
| 4+ | Optic nerve head is obscured |

**Supplementary Table 3. The ASUWOG score for FA**

| Maximum score | Angiographic sign |
| --- | --- |
| 3 | Optic disc hyperfluorescence |
| 4 | Macular edema |
| 7 | Retinal vascular staining/leakage |
| 10 | Capillary leakage |
| 6 | Retinal capillary nonperfusion |
| 2 | Neovascularization of the optic disc |
| 2 | Neovascularization elsewhere |
| 2 | Pinpoint leaks |
| 4 | Retinal staining/pooling |
| 40 | Total |

**ASUWOG=The Angiography Scoring for Uveitis Working Group.**


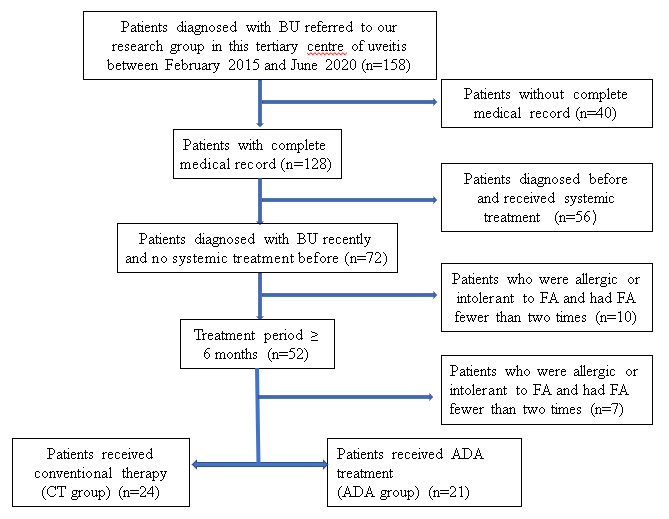


**Supplementary figure 1: Flow diagram of the selected inclusion criteria for BU patients admitted to this tertiary centre for uveitis.**
